# The Development of a New Model of Educational Leadership: Leadership for Teacher Flourishing

**DOI:** 10.1007/s41463-024-00181-z

**Published:** 2024-06-14

**Authors:** Katy Granville-Chapman, Matthew T. Lee, James Ritchie-Dunham

**Affiliations:** 1 Department of Education, University of Oxford, 5 Norham Gardens, Oxford, OX2 6PY UK; 2 Institute for Studies of Religion, Baylor University, One Bear Place #97236, Waco, TX 76798 USA; 3 McCombs School of Business, University of Texas–Austin, 2110 Speedway, Austin, TX 78712 USA

**Keywords:** Flourishing, Leadership, Love, Teachers, Schools

## Abstract

This paper contributes to a broader movement in which the telos of leadership is flourishing, and the primary role of a leader is to promote the flourishing of their team members through creating a loving environment. In support of this, we propose a new perspective on, and associated model, of educational leadership: ‘leadership for teacher flourishing’ (LFTF). This model was developed through a literature review and a mixed methods research project across 78 British schools with collaborative and participatory elements which asked how school leaders could improve the flourishing of teachers. The quantitative and qualitative findings suggested that key factors in enabling teachers to flourish, and therefore incorporated into the model ‘LFTF’, were positive relationships; opportunities for growth; positive impact on subjective wellbeing; and increasing teachers’ sense of meaning at work. The ways leaders could impact these factors were reported as: being supportive and compassionate; being trustworthy; giving teachers autonomy; enabling teachers to grow; being appreciative and focusing on strengths; and enabling teachers to do meaningful work. It was also found that not only leaders could influence flourishing; teachers with no formal leadership role could also positively impact teacher flourishing. It is suggested that virtuous dispositions in leaders are required to ensure they are consistent in acting in ways that promote flourishing. We argue that this integration of virtue and a desire to promote flourishing is properly understood as love. The participatory and collaborative phase of the research showed that teachers wanted autonomy in deciding what would help them to flourish. The conclusion sets out the implications of this research for policy on the training and selection of school leaders, to improve not only the flourishing of teachers, but also pupils and communities.

## Introduction

This article supports a broader movement which suggests that the primary role of a leader is to create a loving environment in which their teams and the community can flourish. This has been termed ‘leadership for flourishing’ (Ritchie-Dunham et al. Forthcoming) and ‘love in action’ (Ritchie-Dunham et al. In Press). Philosophers argue for the intrinsic value of flourishing (Aristotle 2009; Brighouse 2006; Kristjánsson 2013; Macintyre and Dunne 2002). We could argue that its intrinsic value is enough justification for flourishing being the primary aim of leadership, especially in light of data showing that millions of people around the world are not thriving or flourishing at work, and also due to the fact that many positive outliers exist across all cultures that have been studied (Clifton 2022; Ritchie-Dunham 2024). We posit that leadership makes a critical difference and that leadership for flourishing in the education sector might offer useful lessons for all sectors, partly because teachers seem to be lagging other professions on important well-being outcomes. If some progress has been made in leading teachers towards greater flourishing, as our study suggests, then there is hope for other sectors as well. Furthermore, over the past decade the significance of flourishing has also been increasingly acknowledged by researchers, health practitioners, and the general public (Su et al. 2014; Levin 2020, 2021). In addition to being an end in itself, cross-sectional, longitudinal and experimental studies have also shown that high levels of flourishing are associated with a variety of positive outcomes, such as: effective learning (Lindorff 2020); productivity and creativity (Huppert 2009); good relationships (Diener et al. 2010); pro-social behaviour (Lyubomirsky et al. 2005); and good health and life expectancy (Chida and Steptoe 2008).

This project focuses on education as a case study in order to discover how school leaders could improve the flourishing both of teachers and leaders. It argues that school leaders have the opportunity to influence the flourishing of teachers, and that this is a worthy goal of school leadership. Whilst there has been a proliferation of research into school leadership and the development of school leaders over the past twenty years, there is a prominent gap in the literature concerning how leaders can improve the flourishing of teachers.

### Current State of Teacher Flourishing and Impact on Their Students

Teachers are not all flourishing. School teaching is recognized globally as one of the most stressful jobs (Lhospital and Gregory 2009; Kyriacou 2000). Johnson et al. (2005) in a comparison of 26 diverse occupations within the UK found that teachers score below average on physical health, psychological well-being and job satisfaction. Johnson et al. (2005) also found that teachers have an increased risk of burnout and a higher prevalence rate of psychosomatic complaints. In addition, the number of therapeutic treatments of stress-related disorders, such as depression in teachers is growing (Austin et al. 2005). The COVID-19 pandemic has only exacerbated these concerns with over half of teachers participating in a YouGov survey reporting that they were experiencing anxiety and nearly a third saying they felt hopeless (YouGov 2020). Researchers and educators have described the harmful impact of teacher stress in schools and the damage it creates in both the education system and society at large (Carlyle and Woods 2002). Furthermore, it is argued that teachers cannot transmit emotional and social competence and well-being effectively to their students if their own emotional and social needs are not met (Weare and Gray 2003).

Teaching is considered to be a key contributor to a flourishing society (Arthur et al. 2015). Teachers will work with several hundred, if not thousands, of students over their careers, influencing the lives of those students and indirectly impacting the lives of those connected to their pupils. This project begins with the premise that, if school leaders can enhance teachers’ flourishing, then this will also have a positive impact on the students they work with, as well as on the teachers themselves.

The primary aim of the analysis of these data was to identify trends that could be easily understood and potentially applied by school leaders and teachers in their contexts, and with broader relevance to leaders outside of the education sector. This was done through calculating and comparing percentages across the items and 5 ratings options. Participants were also asked how important they perceived each dimension was to their flourishing.

### The Relevance of a Focus on School Leaders to Teacher Flourishing

Whilst there is very limited research into the impact of school leaders on teacher flourishing, there is evidence to suggest that leaders across sectors can influence the flourishing of those they lead. For example, Kuoppala et al. (2008) conducted a systematic review and a meta-analysis of 109 articles and presented results from 27 papers from Europe, the USA, Australasia, Asia and Africa. They conclude that good leadership seems to improve flourishing at work. Kuoppala et al. (2008), however, call for more well-founded, rigorous prospective studies to strengthen and clarify the evidence concerning the relationship between leadership and employees’ flourishing at work. This project hopes to contribute to reducing this gap in the literature in one sector, in the hope that our process and findings might be replicated in other sectors.

This project drew upon work from positive psychology: the scientific study of ordinary human strengths and what goes well in life. Positive psychology research focuses on learning what the nature is of the effectively functioning individual or organization, which successfully adapts and learns (Peterson 2006; Sheldon and King 2001). From the positive psychology movement, three other particularly relevant fields for this research have emerged: humanistic management; positive education; and positive organizational scholarship (including positive leadership). The project also drew from, and hoped to contribute to, the educational leadership literature and the general leadership literature; both of which implicitly consider flourishing, but rarely consider explicitly the role of leadership in flourishing.

### Defining Key Terms

Flourishing in this project was defined using participants’ understanding of the term, which was put forward in the initial pilot focus group and resonates with but simplifies many of the definitions of flourishing in the literature: ‘*being at your best*’ (for example, VanderWeele 2017; Fox 2013; Butler and Kern 2016). Flourishing is, however, recognized as a multi-dimensional construct and this project focuses on teachers’ work-lives. This project focused on exploring where and how leaders can improve the following dimensions of teacher flourishing in their work contexts: relationships; subjective wellbeing (SWB); engagement; meaning; mastery; autonomy; and optimism, drawing upon Su et al. (2014) model of psychological wellbeing and associated Comprehensive Inventory of Thriving. These domains can be expanded to encompass Ecosystem-Wide Flourishing (Ritchie-Dunham et al. In Press), but it is helpful to begin the exploration of flourishing with a less comprehensive and more manageable set of domains (Lee and Wellinghoff In Press). A school leader, within this project, was anyone working in a school with a named, formal leadership position and with responsibility for one or more members of staff. This definition included middle leaders (such as heads of department and heads of year); senior leaders (including deputy heads and assistant heads); head teachers; and executive head teachers. It was shown in this research project, however, that teaching staff, whatever their position or role could have an impact on others’ flourishing; any member of staff could improve their own and others’ flourishing if given the opportunity and support. Teachers were defined as any member of staff with a teaching role.

In an educational setting, the desire to promote flourishing—and the ability to actually do this with effectiveness and practical wisdom—transforms education into a *love-based technology of change* that can impact population health and wellbeing (see Lee et al. 2021; Levin 2023; Maparyan 2012). The word ‘love’ will seem out of place to those who have internalized the many cultural confusions (Reeve 2005) that have obscured the power and clarity of this greatest of all ideas (and ideals). But love, properly understood, has been declared the essence of flourishing (Briggs and Reiss 2021) and this idea has guided educational practice (Lee and Pearson In Press). It involves the recognition that we learn in relationships, but not just any kind of relationship. As Lee et al. (2021, p. 204) have framed it, in educational contexts possibilities for “transformation and flourishing are connected to ‘feelings of being loved and loveable,’ on the one hand, and the development of personal agency in the context of democratic practices.” This requires the creation and regeneration of a social container that orients human beings toward seeking each other’s greatest good. In other words: a social space organized by a “logic of love” (Guillén 2021, p. 149).

In common usage, to love well means to relate to the other in healthy ways and contribute to the good of the other (VanderWeele 2023; Lee 2022). Self-love would entail relating to one’s own best possible self and drawing it forth in ways that promote deepest self-flourishing. Some philosophers and theologians (e.g., Aquinas, Frankfurt, Tillich, respectively) have claimed that “all of our reasons for action are grounded in love,” that love is “the cause of all the various passions,” that there is “no higher principle” than love (see VanderWeele 2023, p. 50; Lee 2022, p. 11). Indeed, healthy expressions of love are at the heart of a truly humanistic form of leadership, one that refuses to dehumanize and instead works skilfully for the flourishing of people and planet (Pirson 2022; Lee 2022; Hummels et al. 2021, 2022; Guillén 2021; Ritchie-Dunham et al. Forthcoming).

### What Does the Leadership Literature Say About Flourishing?

There is scarce research into the influence of school leaders on the flourishing of teachers. Further, there is limited consideration of the influence of leaders on the flourishing of those they lead in the general leadership literature, with only a small number of studies focusing on the effect of leadership on flourishing. The literature review examined the most prevalent models of both school leadership, and general leadership models, and the extent to which these theories provide information on how leaders applying these models could influence the flourishing of the teachers they lead. The review considered instructional, student-centred, distributed, transformational and moral leadership to assess what these theories of school leadership said about the flourishing of teachers. The review chose to explore these theories as they are frequently cited in the literature (Gumus et al. 2018) and have the potential to influence teacher flourishing. It then reviewed theories that reside beyond the education literature and their potential implications for teacher flourishing, focusing on what responsible leadership (Maak and Pless 2006; Waldman and Galvin 2008), humanistic management (Arnaud and Wasieleski 2014; Melé 2016), servant (Greenleaf 2002; Farling et al. 1999), positive (Cameron 2012), and authentic leadership (Avolio and Gardner 2005) and followership theories (Shamir 2007) said about flourishing.

The school leadership literature presented several theories of leadership that have the potential to influence the flourishing of teachers indirectly; none of the theories reviewed, however, included the flourishing of teachers as a stated aim. The theories also did not include the dimensions of flourishing that this project focuses on as defined outcomes of the leadership models (relationships; subjective wellbeing (SWB); engagement; meaning; mastery; autonomy; and optimism, from Su et al. 2014). Furthermore, research into these models has focused more on their impact on school performance than on their influence on human flourishing.

Within the general leadership literature only humanistic management and positive leadership stated flourishing as an explicit goal. Some aspects included in the general leadership models and theories reviewed had the potential to improve the flourishing of teaching staff if applied by school leaders, but within the school leadership literature the mechanisms for this influence were not explored explicitly. In the leadership literature, the impact of responsible, servant, authentic and positive leadership, as well as humanistic management on flourishing was more explicitly reported. The literature review revealed a research gap in both the school and general leadership literature concerning theories of leadership that directly address the flourishing of teaching staff.

## Methods

This project began with the aim of discovering how school leaders could improve the flourishing of teachers; to do this, data were collected on three research questions:

- RQ1. What do teaching staff (including school leaders) understand by the term ‘flourishing at work’?
- RQ2. What do teaching staff believe school leaders could do to improve their flourishing?
- RQ3. What kinds of strategies could school leaders adopt with the intention of improving the flourishing of teaching staff?

These questions were answered using a mixed-methods approach with collaborative and participatory elements.

Table 1 presents the data sets that were used to answer each of the research questions. Table 2 shows the data collected during each of the phases of the research.

**Table 1:** How each data set was used to answer the research questions

Research questions	Data collected
1. What do teaching staff (including school leaders) understand by the term ‘flourishing at work’?	1. Focus group session 1 exploring what participants understood by the term ‘flourishing’ in their work
	2. Online questionnaire: Likert-type rating questions asking the importance of seven dimensions of flourishing
2. What do teaching staff believe leaders could do to improve their flourishing?	1. Online questionnaire: open questions
3. What kinds of strategies could school leaders adopt with the intention of improving the flourishing of teaching staff?	1. Focus groups 2–4 exploring opportunities for interventions to improve the flourishing of teaching staff
	2. Online survey in School 1 plus focus groups and individual interviews in Schools 2 and 3.

**Table 2:** An overview of data collection and how methods were mixed

Phase	Data collected
Phase 1	Pilot exploratory focus group to design Questionnaire 1
	Pilot exploratory focus group to design Questionnaire 2
	Pilot of the online questionnaire
Phase 2	Exploratory online questionnaire. Used for RQ1, RQ2 and RQ3
Phase 3	Pilot focus group 3 to design focus group questions and delivery
	Pilot focus group 4 to design focus group questions and delivery
Phase 4	Focus group session 1 (with 3 × schools). Used for RQ1 and RQ2
	Focus group session 2 to design intervention. Used for RQ3
Phase 5	Implement interventions (in 3 × schools). RQ3
	Focus group session 3 to reflect on progress of intervention. RQ3
	Survey and interviews about the impact of interventions in School 1. RQ3
Phase 6	Focus group session 4 (with 3 × schools) to reflect on the impact of interventions. RQ3

RQ1 was answered through the collection of quantitative and qualitative data, which were used to assess the extent to which various dimensions of flourishing were relevant to teachers’ and school leaders’ understanding of flourishing. These data were collected through an online questionnaire, using Likert style ratings, as well as open questions, and the first of the series of focus groups with three schools. The online questionnaire was answered by teaching staff in all roles in 48 state-funded secondary schools, 17 state-funded primary schools, 8 fee-paying secondary schools and 5 fee-paying primary schools in the UK. Therefore, a total of 78 UK schools participated. The respondents included 98 teachers with no named leadership responsibilities, 6 head teachers, 70 middle leaders (heads of department, subject leaders, heads of year or housemasters/mistresses/parents)^1^ and 20 senior leaders 2. A total of 194 teaching staff participated and the completion rate was 94%, where the questionnaire was started.

The questionnaire drew upon the items included in Su et al.’s (2014) Comprehensive Inventory of Thriving (CIT), but these were adapted to focus on the impact of leaders on dimensions of teacher flourishing. Qualitative data were also collected to provide a richer understanding of the data, as flourishing is such a complex concept and varies significantly between individuals. Qualitative data were collected through open questions on the online questionnaire and the first focus group discussions in three UK schools, where participants were asked to describe their understanding of flourishing in a work context.

RQ2 also required quantitative and qualitative data (for similar reasons). It was useful for school leaders and policymakers to be given the trends in people’s answers regarding what they believed leaders could do to impact their flourishing; richer data was also required, though, as this question asked what school leaders could do to improve flourishing. Data were collected for RQ2 in the online questionnaire and first focus group discussions.

RQ3 aimed to find out what improvements in flourishing could look like in practice in a school setting, rather than solely in theory. To answer this RQ, participants in the three case study schools were asked to design and deliver interventions collaboratively to improve the flourishing of teachers in their schools. This process began in the second focus group where teachers were asked to consider the questionnaire data and then suggest and decide on two strategies that their school could adopt to improve the flourishing of teaching staff. The third focus group was used to allow the groups to reflect on how the intervention was progressing and whether they wanted to make any changes to the intervention. The final session was an opportunity to reflect on how effective their strategies had been in improving flourishing in their schools. Further data were collected through an additional online survey for School 1 and from the WhatsApp group for School 3. They reviewed the impact of these interventions, as well as refining them, halfway through the project; they evaluated their impact in the final series of focus group discussions.

A participatory (Bergold and Thomas 2012) and collaborative approach (Hafernik et al. 1997) was therefore introduced at this point. This allowed teaching staff and school leaders to consider the data collected in RQ1 and RQ2, and then decide what they wanted to try in their own contexts. This provided the opportunity to explore the leadership processes and challenges that arose when leaders and teachers worked collaboratively to improve the flourishing of teaching staff. It also began to bridge a perceived methodological gap between ‘academic’ research and ‘practitioner’ research (Jagosh et al. 2012).

The research was influenced by pragmatism, specifically Tashakkori and Teddlie’s (2003) interpretation of pragmatism, which ‘focuses on “what works” as the truth regarding the research questions under investigation’ (p. 713). It also drew on Dewey’s (1938) ‘problem-centred pedagogy’ and notion that democracy is about more than just votes; rather, it is the idea that all societal institutions should aim to promote flourishing: something this project is trying to grapple with for schools. This research took a virtuous approach to ensure it was ethical. The research showed respect for participants by enabling them to voice their thoughts on the quantitative and qualitative data that had been collected, as well as providing further information on what enabled them to flourish. It also empowered participants to design interventions that would improve their own and others’ flourishing. The authors have named this methodology ‘loving collective inquiry’. The participants co-created a loving environment from which to inquire together about what would most effectively enable the members of their school community to flourish.

## Results

This section presents the findings to RQ1: What do teaching staff understand by the term flourishing at work?; RQ2: What do teaching staff believe school leaders could do to improve their flourishing? and RQ3: What are the impacts of the collaborative creation of interventions that aim to improve the flourishing of teaching staff?

### Results: RQ1: What Do Teaching Staff Understand by the Term ‘Flourishing at Work’?

Su et al.’s (2014) dimensions of psychological wellbeing (relationships; subjective wellbeing (SWB); engagement; meaning; mastery; autonomy; and optimism) were found to be helpful as a means of organising a multifaceted and complex phenomenon; further, there was adequate fit with the data to justify their continued use as a means of understanding how teachers understand flourishing. See (Granville-Chapman 2021) for statistical validation of the survey instrument. Each of the dimensions of Su et al.’s (2014) model of psychological wellbeing was found to be relevant to teachers’ understanding of flourishing, and these factors were difficult to isolate, with many of the teachers’ responses covering more than one dimension. The dimensions were also frequently described as overlapping and affecting one another in the literature (Su et al. 2014). The data also suggest that relationships are particularly important in teachers’ understanding of flourishing, which serves as a reminder that love is a key: not just any relationships will suffice. As we will see, relationships that express and nurture the deepest human needs (Ryan and Deci 2000; see also Waldinger and Schulz 2023) are required.

### Results: RQ2: What Do Teaching Staff Believe School Leaders Could Do to Improve Their Flourishing?

This RQ aimed to explore what teaching staff believe leaders could do to improve their flourishing. Data were collected through the online questionnaire’s open questions and the focus group discussions with the groups from three different UK schools. What had been shown so far in the project was that relationships in the literature, and in the perception of participants, were important to people’s understanding of flourishing. Relationships were also shown to be linked to all the other factors involved in flourishing that had been explored to this point. The centrality of relationships is not surprising, and it reminds us of the conclusion drawn by the director of the longest longitudinal study of human well-being in history: “Happiness equals love. Full stop.” (Vaillant, quoted in Shenk 2009). Or as the current study director put it, “if we had to take all eighty-four years of [the research] and boil it down to a single principle for living… it would be this: *Good relationships keep us healthier and happier. Period*” (Waldinger and Schulz 2023, p. 10). As for RQ1, it was found that there were many dimensions of flourishing that overlapped, and participants had different perceptions of what would most influence the extent to which they were flourishing. The factors most frequently reported that participants thought leaders could influence to improve their flourishing were, using their words, rather than Su et al.’s (2014) dimensions, positive relationships, time/balance, growth,^3^ and subjective wellbeing.

### Results: RQ3: What Are the Impacts of the Collaborative Creation of Interventions that Aim to Improve the Flourishing of Teaching Staff?

Focus groups of teaching staff in three schools reflected on the impact of trial interventions, approximately halfway through and at the end of the intervention. See (cite dissertation) for protocol and assessment details. The interventions that schools discussed in depth and/or implemented were:

- School 1 (an independent secondary school in Southeast England): yoga, coaching and failure stories.
- School 2 (a secondary academy in Southeast England): positive boards in departments, discussion of behaviour and email management.
- School 3 (a secondary Academy in Southwest England): WhatsApp group and pay it forward acts of kindness.

Within these, participants reported the generalised positive impacts on their flourishing shown in Table 3.

**Table 3:** The impacts of the interventions on factors most frequently identified by teaching staff as affecting participants’ flourishing

Intervention	Impact on factors identified as influencing teaching staff flourishing
Yoga	Positive relationships Growth SWB and optimism Appreciation + gratitude Physical health
Coaching	Positive relationships Growth SWB
Positive boards	Positive relationships SWB Appreciation + gratitude
WhatsApp group	Positive relationships SWB
Pay it forward kindness acts	Positive relationships SWB

As shown in Table 3, participants reported that the development and formation of positive relationships (a key ingredient in love) were affected positively in all interventions. Both a decrease in stress and an increase in positive feelings were also reported for each of the interventions. Yoga and coaching were reported to have enabled participants to grow. This mirrored the findings for the earlier questions, whereby positive relationships, SWB and growth were perceived to be particularly important to participants’ flourishing. Gratitude and physical health were also reported benefits of the yoga intervention, with gratitude also cultivated in the ‘positive boards’ intervention.

This article proposes that school leadership involves having a positive impact on the flourishing of others, and that the role of school leaders in promoting the flourishing of teachers is particularly important. Empirical evidence from surveys, focus groups, and pilot interventions support the impact of this shift in school leadership focus towards leadership for teacher flousing and flourishing-focused interventions. These results suggest that perhaps the most important method school leaders can utilise to improve the flourishing of those they lead is through giving their teams the opportunity to co-create and co-lead interventions to improve their own and others’ flourishing. This has implications for other sectors and is actually a reasonable shorthand definition of (organising) *love*. Our project’s findings suggest that what the interventions are (in this project, yoga, coaching, positive boards, WhatsApp groups and random kindness acts)—is less significant than the process of empowering teachers to co-design activities with other teachers that they perceive will benefit the staff, and then enabling teachers to lead them, evaluate their impact, and suggest and deploy improvements. The interventions chosen by the teachers in this project were diverse and targeted different aspects of flourishing. For example, yoga would traditionally be considered for focusing on improving an individual’s physical health and mental wellbeing; whereas acts of kindness and coaching focus on helping others to flourish. Nonetheless, there was overlap in the impact on domains of flourishing that were affected by the intervention.

## Discussion: A New Model of School Leadership: Leadership for Teacher Flourishing

The overarching aim of this research was to explore how school leaders could improve the flourishing of teachers, and it led to the proposal of a new model of school leadership, leadership for teacher flourishing (LFTF), to add to current models of teacher, school, and educational leadership. The model is summarised in the table below (Table 4).

**Table 4:** Model of school leadership: leadership for teacher flourishing (LFTF)

Preconditions of love in processes of leading and/or organizing for flourishing	Examples of enabling virtuous dispositions of leaders	Outcomes of the processes of leading and/or organizing that express love and result in flourishing
Being supportive and compassionate. Ask teachers what support and compassion they need and would value. Acting to meet those requests and needs, while balancing the needs and interests of all stakeholders.	Supportive, compassionate, caring, loving, selfless	Positive relationships SWB
Trustworthiness Ask teachers how they could most effectively build trust within the team and/or school. Showing integrity, being compassionate and supportive, empowering teachers	Integrity, compassion, fairness, generosity, gratitude, and courage.	Positive relationships SWB
Giving autonomy/empowering Understand what autonomy the teacher wants in line with their strengths and capability. Work with teachers to maximize autonomy within the constraints of (possibly externally imposed) accountability measures.	Empowering, trusting, positive, humility, understanding	Positive relationships SWB Growth Meaning
Enabling teacher growth Ask teachers how they would like to grow and develop and help facilitate and support this growth	Empowering, trusting, supportive, positive	Positive relationships SWB Growth Meaning
Appreciativeness and focus on strengths Find out how teachers like to be appreciated and designate time and resources to this. Take the time to learn what teachers’ strengths are and recognize and celebrate these	Gratitude, empowering, trusting, supportive, positive	Positive relationships SWB
Enabling teachers to focus on meaningful work Remove all work that is not directly helping pupils to achieve their potential. Help teachers to connect to aspects of their work that are meaningful, for example, improving pupil outcomes. Help teachers job craft to make their work feel more meaningful.	Empowering, trusting, supportive, positive, understanding, purposeful, visionary	Positive relationships SWB Meaning

Love is implicated in all of these ways. In fact, they can be seen as forms of love in action. The model focuses on how leaders could improve the flourishing of teachers. It includes possible actions leaders could take and examples of virtuous dispositions that could improve the influence of leaders on teacher flourishing. The model also highlights the most frequently reported dimensions of flourishing that teachers perceived that school leaders could influence that would have an important impaction on their flourishing. The discussion elaborates on what the new model (LFTF) may mean in a school context and connects this project’s findings to other research on the topic. The project also proposes a new dimension of leadership, which is not included explicitly in other models of school leadership, or in the school leadership literature: ‘*supporting and promoting the flourishing of others*.’

The LFTF model is intended to contribute to filling a gap in the literature concerning the influence of school leaders on the flourishing of teachers. This model is based on a new approach that suggests effective school leadership prioritizes the flourishing of teachers; this, in turn, evidence suggests, improves pupil outcomes (for example, Sammons et al. 2007). LFTF propose that flourishing should be a key focus for leaders because of its intrinsic worth (Kristjánsson 2013); and because of its performance benefits (for example, Cameron 2012). This project also found, though, that leadership and having a positive impact on flourishing was not only possible for someone in a formal leadership role. In fact, it was also shown that teaching staff at any level of an organization could influence the flourishing of others; this finding echoed some of the later literature on leadership, which views leadership as service and impact on others, rather than an allocated role or the achievement of change or results (for example, Laloux 2014). The model has particular congruence with models of responsible, servant and positive leadership, but takes each of these models further with its inclusion of specific actions and dispositions that emerged as being influential in flourishing (Maak and Pless 2006; Patterson 2003; Cameron 2012). Responsible, servant and positive leadership have also all been shown to have positive impacts on follower flourishing (Maak and Pless 2006; Patterson 2003; Cameron 2012).

The influence of school leaders on flourishing was much less commonly explored in the school leadership literature, which seems to focus more on the impact of school leaders on pupil outcomes (attainment) and a focus on strategy and operations rather than on teacher or, indeed, pupil flourishing (for example, Earley et al. 2012). Harris and Muijs (2002) observed that, while there is considerable evidence of the positive effects of teacher leadership, there is limited research on the nature of teacher leadership, adding that there is a need for both empirical evidence of teacher leadership in action and for different models of teacher leadership. Gumus et al. (2018), also cite this proposal made by Harris and Muijs (2002) and, following a systematic review of studies on leadership models in educational research from 1980 to 2014, say that the development of new educational leadership models can be explained as researchers’ efforts to clarify the definition and practices of effective leadership from different perspectives. This project’s proposed new model (LFTF) aims to add to this understanding through suggesting effective leadership actions and virtuous leader dispositions for improving the flourishing of teachers.

The key challenge leaders in all organizations face, however, when organizing for love and flourishing, is the need to take decisions. The factors included in the model LFTF address what kind of style of leadership is required. In line with the leadership style that emerges from these virtuous dispositions, the decision-making itself should ideally reflect balanced decision-making that weighs all the needs and interests of the stakeholders (pupils, staff, parents, the wider community). The leadership should also provide adequate and timely feedback to the stakeholders explaining how their flourishing has been considered; and creating a responsive and accountable culture: accountability should be focused on the request for every member of the organization to support and promote each other’s flourishing; the responsiveness appreciates that what stakeholders need to flourish will change over time.

## Virtuous Dispositions and Actions

The main ways identified in this project that leaders could impact flourishing were through: being supportive and compassionate; being trustworthy; giving autonomy, or empowering teachers; enabling teachers to grow; being appreciative; and enabling teachers to focus on meaningful work. For a leader to successfully carry out these actions it is suggested that virtuous dispositions are also required to ensure the leader is consistent in acting in ways that promote flourishing. The specific virtues a leader needs to influence positively the flourishing of teachers will, however, vary depending on their relationship and the context. Furthermore, a key recognition the LFTF model makes is that, when articulated in words or actions, virtuousness tends to be interpreted as one or more discrete virtues; the attributing of virtues to words or actions is arguably, though, a subjective exercise (Newstead et al. 2019). For example, a school leader acts on good inclination (acts on virtue) and covers a teacher’s lesson so that they can watch their child’s nursery school play. Some people may consider this to be indicative of the virtue compassion; while others would say it is self-sacrifice, generosity, or humanity. The model does not, therefore, propose that it is possible to say that certain virtues are the most important for school leaders since the context will constantly vary and the articulation of virtues into words or actions is also subjective. It does, however, suggest virtues that were shown to be likely to predict the leader actions identified by the model that could be beneficial to teacher flourishing.

The LFTF model builds upon research into the dispositions of leaders and the impact of this on flourishing (e.g. Hannah and Avolio 2011). Bair (2017) says that a review of the literature on dispositions revealed that in spite of a lack of consensus on a precise definition for the term, researchers predominantly describe dispositions as internal attributes or psychological characteristics that motivate action, or as a propensity to act in a certain way that tends to be predictive of future action (Borko et al. 2007). Hollon et al. (2010), however, describe this as a choice as opposed to a tendency: ‘*the choice to act or react in characteristic ways in certain situations*’ (p. 123). Furthermore, Sockett (2009) claims that while dispositions are related to action, they only predict acts, rather than causing them.

For the model LFTF, dispositions are defined in a similar way to Hollon et al. (2010) as a choice to act or react in a characteristic way, thus highlighting the agency of school leaders to choose to act virtuously. Furthermore, this paper agrees with Diez (2006) who says that a disposition is the regular expression of attitudes in stable behavioural patterns. This thesis proposes that the choice to act virtuously in a consistent manner is related to school leaders’ attitudes; thus, if a school leader has a compassionate attitude, they are then likely to choose to act compassionately. For this to be a disposition, the leader would act compassionately consistently. The model, LFTF, uses the term disposition, but with the appreciation that for a school leader to habitually act virtuously could be even more effective in promoting flourishing since some scholars argue that habits can be performed without conscious attention, whereas dispositions are intentional and voluntary (Bair 2017). A virtuous habit such as compassion could be less effortful than merely a compassionate disposition for a stretched and stressed school leader.

Villegas (2007) argues that dispositions also have a social component; she proposes that they are the tendency to act in a certain way, under particular circumstances and are strengthened or weakened through experiences with others. Thus, reinforcing the idea that dispositions are not static traits; they are likely to develop as people encounter experiences and opportunities (Feiman-Nemser and Schussler 2010). This suggests that the dispositions of leaders are influenced, and influence, the dispositions of others, implying that relationships and cultural factors are important in the development of dispositions. While this paper does not focus on the cultural influences within schools on teacher flourishing, as it concentrates on the direct influence of individual leaders (whether or not in a named role) on teachers, it does recognize the importance of a loving culture and environment on the development of character and, therefore, flourishing. This is an area recommended for future study.

In the review of the literature, it was found that a variety of virtues were mentioned in relation to the moral component of several leadership theories, including moral and ethical, responsible, servant, transformational, and positive leadership, as well as humanistic management. This paper builds upon Cameron and Caza’s (2003) work which says that virtuousness goes beyond concepts such as ethics or moral reasoning: ethics and moral reasoning focus on what is sufficient, necessary or instrumental; whereas, virtue goes beyond this and focuses on the highest human potential, or flourishing. Enabling teachers to reach their highest human potential is a worthy goal of school leaders and this thesis proposes that the virtuous dispositions in the model LFTF go some way towards enabling this goal.

The discussion will now consider in more detail each of the ways included in the model LFTF that leaders could improve the flourishing of teachers, drawing upon the main lessons learnt from the data collected for each of the three research questions and the literature.

## The Proposed Model, ‘LFTF’, in Practice

For the model to work in practice it is suggested that the first thing a leader must do is gain an excellent understanding of the teachers, or people they lead. The leader needs to gain a full appreciation of how teachers want to be supported and to have compassion shown to them. As was seen from the diversity of responses coded into this category, individuals vary significantly in their preferences around how a leader could show support and compassion to them to enable them to flourish. Also of note, empowering teachers and giving them autonomy were other factors identified by teachers and leaders as being important for them to flourish: how much autonomy and how this autonomy is given, as well as how much support is needed also varies between individuals. This paper, therefore, recommends that a leader asks teachers directly how they can best support them, show them compassion, empower them, and give them autonomy; then act on the teacher’s requests, where this is possible, given the demands on leaders to balance a range of stakeholders’ needs. Where it is not possible to meet a teacher’s needs, a leader will explain the reason for this; this will perhaps be the most challenging aspect of leadership for someone who wishes to support and promote flourishing and to create a loving environment. Equally, literature suggests that there are important differences between individuals’ strengths; what one teacher is particularly strong in, is unlikely to be the same as another teacher’s forte. An individual’s strengths can also vary across time and context. To promote teacher flourishing, and therefore performance, a school leader needs to know what a teacher’s strengths are (and continue to improve their understanding of this throughout their leadership) in order to focus on those and allow teachers to do work that uses those strengths (Peterson and Seligman 2004). Guessing or generalising is not likely to be effective in enabling teachers to flourish. This, the data showed, is true also of how teachers want to grow and develop, as well as how teachers wish to be shown appreciation and gratitude.

Furthermore, to enable teachers to focus on meaningful work, a leader needs to know what individual teachers value. Asking them directly what they value or working with teacher to uncover their values and purpose through coaching, was shown by this project to be effective in enhancing teachers’ sense of meaning at work. It is, therefore, suggested, that the key method a leader can translate the LFTF theory to practice is to get to know those they lead extremely well. As mentioned above, coaching is a good method for this, but questions directed towards enhancing a leader’s knowledge of each of the areas mentioned above are also necessary.

This proposal reflects the core philosophy of a book a colleague and one of the article’s published which can be summarised as ‘know your people; love your people; inspire your people’ (Granville-Chapman and Bidston 2020). In the book, we argue that only by knowing your people can you show them love; and only once you know your people and have shown them love can you inspire them through enabling them to connect to and be active in areas that are meaningful to them. The book draws upon the business literature and interviews with leaders from a range of professions including (amongst others) the law, sport, the charity sector, technology, politics, health, and the military.

This paper argues that the application of the model LFTF by school leaders, particularly through finding out the extent to which they are exhibiting virtuous dispositions that are relevant to the needs of the individual teachers they lead, would have a positive impact on the flourishing of teachers and, therefore, also pupils. How school leaders develop such virtuous dispositions is beyond the scope of this paper, but the authors are co-leading a project ‘leadership for flourishing’, which hopes to begin to answer this. Having considered the application of the model in practice, this article will now consider the strengths and limitations of the project.

## Strengths and Limitations

The use of mixed methods, whilst it had its limitations, was an appropriate way of accessing two complex concepts: flourishing and leadership. It was beneficial to collect both quantitative and qualitative data. The quantitative data highlighted general trends which could be used by school leaders and policy makers; it identified trends in participants’ understanding of the term flourishing in their work contexts and in what teachers believed school leaders could do to improve the extent to which they flourished in their school roles. The quantitative data was also used by participants in the collaborative phases of the research and enhanced their understanding of what other teachers’ perceptions were. The use of participatory and collaborative research was appropriate for the aims of the project and it was beneficial to involve teaching staff throughout the project to improve the relevance of its design and their engagement with the research. This also bridged a perceived gap between research and practice, and empowered teachers to design interventions to improve flourishing that were relevant to their schools’ contexts.

The online survey provided a broader and larger sample than could have been achieved through focus groups and interviews alone; teachers from 78 schools in the UK in a range of roles completed the survey. The sample for the online questionnaire was not, however, as representative as it could have been for UK schools as the sampling was purposive and then utilised snowball sampling to increase the number of respondents. It is likely that respondents were interested in the topic and, therefore, not wholly representative of teachers in the UK. It would also have been better to have collected a higher number of responses and to have ensured that within each school that responded, teachers in a representative range of roles were included. The size and nature of the sample mean that any generalisation of the findings to other contexts should be approached with caution.

The qualitative data collection methods were helpful for gaining richer data about a complex multidimensional concept, flourishing. They also enabled a more in-depth reflection on the creation of interventions by participants. The qualitative methods could, however, have been enhanced by using more individual interviews to gain a deeper understanding of what individual teachers and school leaders understood by flourishing and what leaders’ impacts could be on flourishing. The selection of the schools involved in the participatory phase of the project was dictated primarily by access, so the schools were within one and a half hour’s drive of the school I work in, and they were schools that I had worked with through our Teaching Schools Partnership. Although the Teaching School does not dictate leadership policy or culture within the member schools, there is some shared understanding between the schools of leadership as all members have sent at least one teacher on a leadership programme that I have run. The three schools are also located in semi-rural areas in the South East of England. It would have been interesting to have included cases from very different contexts, such as schools which are part of a large academy group, or those in an inner-city location. As data on teacher wellbeing increases in its availability, it could also be beneficial to carry out purposive sampling to include schools with varying levels of staff flourishing.

The methods of analysis could have been enhanced by using a team of researchers to check the data and spending more time with participants exploring with them the extent to which the findings and conclusions reported were congruent with what they were intending to contribute to the research.

## Contributions to the Literature

The model proposed by this project contributes to the school leadership, and general leadership literature. The model focuses on the dispositions of leaders and the actions leaders could take to improve teacher flourishing, whilst acknowledging that those dispositions and actions, when enacted by someone not in a named, formal leadership role are also likely to improve the flourishing of those they have a relationship with (as was shown to be the case in the collaborative and participatory phase of the research). The model resonates particularly with models of servant and positive leadership but takes these further with its inclusion of specific dispositions that emerged as being influential in flourishing (Maak and Pless 2006; Patterson 2003; Cameron 2012). Servant and positive leadership have also all been shown to have positive impacts on follower flourishing (Maak and Pless 2006; Patterson 2003; Cameron 2012).

Contributions to the literature also included using an established model of flourishing and modifying it to focus on the impact of leadership on flourishing in a school setting; Su et al.’s (2014) Model of Psychological Wellbeing and their associated scale the Comprehensive Inventory of Thriving had not been used in this way before. It provided an instrument that, when adapted to focus on the impact of leaders on teacher flourishing, was well understood by teachers and shown to be relevant to their contexts. Each of the factors included in the CIT was perceived to be influential in the extent to which teachers flourished and the impact of leaders on each dimension could also be measured in the future using this tool. Further modifications could make the instrument even more useful to exploring the flourishing of teachers in a school context and the impact of school leaders on this.

The research brought the school leadership, business leadership and flourishing, wellbeing, thriving and happiness literature together to contribute to an understanding of an under-researched area: how school leaders could improve teacher flourishing. This provided a new understanding of school leadership and its impacts and led to the proposal of a new model of school leadership: leadership for teacher flourishing (LFTF). Collaborative and participatory research was combined with the collection of quantitative and qualitative data from online surveys, focus groups and interviews, which is unusual methodologically but effective for understanding an under-researched and complex area: the influence of leaders on teacher flourishing. It was found that the actions, dispositions, and decisions of school leaders were shown to influence teacher flourishing, and that through working collaboratively with staff, school leaders can improve teacher flourishing.

## Future Research

A future project could explore the extent to which the model of school leadership proposed by this thesis is applicable in other schools and in different contexts (business; medicine; technology, etc.). The project hypothesises that the model, LFTF, would be applicable because the literature provides evidence to support the leadership behaviours that emerged in the findings as being influential in follower flourishing, as well as contributing to the flourishing of the leader themselves. This research is being carried out by members of the ‘Leadership for Flourishing’ group (Ritchie-Dunham 2024), part of the Human Flourishing Program’s Flourishing Network at the Institute of Quantitative Social Science, Harvard University.^4^

## Conclusion

This paper proposes the LFTF model for people working in education to use to create a positive impact on their colleagues’ and, indeed, pupils’ flourishing. The findings from this project are also, however, relevant well beyond education: the model is founded on the idea that a leader’s primary role is to create a loving, thriving and flourishing environment for their teams whatever the context may be (for the business context, see Chapman and Sisodia 2015; Hummels et al. 2021; Lee and Wellinghoff In Press). To do this, it is imperative to get to know each individual a leader has the privilege of working with extremely well and then use this understanding to provide them with the best support possible; so that they in turn can support their teams, and, in the case of teachers, the young people we educate. In a sense, this involves a new way of organizing in many settings where leadership with love for flourishing is not normal, although it is encouraging to learn from positive outliers (Lee and Wellinghoff In Press; Ritchie-Dunham 2024). Our findings suggest that schools—and potentially all organizations—can become laboratories for flourishing when leaders create and expand rituals that convey to organizational members that they are supported, appreciated, empowered, and encouraged to grow and find deeper meaning in their work, all in a context of trust and compassion. Enhancing the flourishing of those in an organisation is not, however, only the responsibility of those in named leadership positions. Everyone within an organisation, or community, has the opportunity to improve the flourishing of those around them. It is hoped that the findings of this research and associated model will be used by leaders, educators, researchers, and policy makers to make a real and lasting impact on the flourishing of those they are fortunate enough to work alongside and to educate.

## Footnotes

1 A precise breakdown of the different middle leader roles was not recorded in this project.

2 All middle and senior leaders were also teaching classes of students. The headteachers were not teaching lessons.

3 Participants suggested that the word ‘growth’ was a word that better represented a key factor in their flourishing than ‘mastery’ (which was Su et al.’s 2014; dimension). They said that growth to them meant development of their whole person, while they thought that mastery was more related to accomplishment in specified fields.

4 As training was a recommendation that emerged from the exploration of what support school leaders need to enable those they lead to flourish, two of the authors also co-created an online, freely available leadership programme (with Granville-Chapman et al. 2020) which trains school leaders (and people in other sectors) in ‘*leading and flourishing in difficult times*’.

## Declarations

### Data Availability

Data on which this article is based can be obtained from the authors upon request.
